# Prediction of Chinese clients’ satisfaction with psychotherapy by machine learning

**DOI:** 10.3389/fpsyt.2023.947081

**Published:** 2023-01-19

**Authors:** Lijun Yao, Ziyi Wang, Hong Gu, Xudong Zhao, Yang Chen, Liang Liu

**Affiliations:** ^1^Clinical Research Center for Mental Disorders, Shanghai Pudong New Area Mental Health Center, School of Medicine, Tongji University, Shanghai, China; ^2^Shanghai Key Laboratory of Intelligent Information Processing, School of Computer Science, Fudan University, Shanghai, China

**Keywords:** psychotherapy, therapy satisfaction, online survey, machine learning, prediction model

## Abstract

**Background:**

Effective psychotherapy should satisfy the client, but that satisfaction depends on many factors. We do not fully understand the factors that affect client satisfaction with psychotherapy and how these factors synergistically affect a client’s psychotherapy experience.

**Aims:**

This study aims to use machine learning to predict Chinese clients’ satisfaction with psychotherapy and analyze potential outcome contributors.

**Methods:**

In this cross-sectional investigation, a self-compiled online questionnaire was delivered through the WeChat app. The information of 791 participants who had received psychotherapy was used in the study. A series of features, for example, the participants’ demographic features and psychotherapy-related features, were chosen to distinguish between participants satisfied and dissatisfied with the psychotherapy they received. With our dataset, we trained seven supervised machine-learning-based algorithms to implement prediction models.

**Results:**

Among the 791 participants, 619 (78.3%) reported being satisfied with the psychotherapy sessions that they received. The occupation of the clients, the location of psychotherapy, and the form of access to psychotherapy are the three most recognizable features that determined whether clients are satisfied with psychotherapy. The machine-learning model based on the CatBoost achieved the highest prediction performance in classifying satisfied and psychotherapy clients with an F1 score of 0.758.

**Conclusion:**

This study clarified the factors related to clients’ satisfaction with psychotherapy, and the machine-learning-based classifier accurately distinguished clients who were satisfied or unsatisfied with psychotherapy. These results will help provide better psychotherapy strategies for specific clients, so they may achieve better therapeutic outcomes.

## Introduction

Psychotherapy is regarded as an approach in which professionally trained clinicians inspire and facilitate changes in the perspectives, emotions, and behaviors of clients using guided conversations and special techniques (1). To date, psychotherapy has proven effective for clients or patients with various clinical complaints, such as depression, anxiety, obsessive-compulsive disorder, alcohol abuse, personality disorder, and children’s mental health complaints (2, 3). However, previous studies have indicated that not all clients were satisfied with psychotherapy, and many factors may influence clients’ responses to psychotherapy (4, 5).

Ignoring the factors affecting client’s satisfaction with psychotherapy may generate many problems. For example, some clients with special complaints, certain ages or occupations, specific education levels, or economic conditions may not be suitable for certain types of psychotherapy (6). The lack of target clients, clinical complaints and theoretically therapy-oriented practices may lead to excessive energy consumption for clients and practitioners (7). Therefore, research that clarifies factors predicting client response to psychotherapy may contribute significantly to the design and improvement in clinicians’ daily interventions. It may potentially reduce the time and economic costs related to psychotherapy for both clinicians and clients (5, 7).

Previously, efforts to predict clients’ responses to psychotherapy have focused on theoretically motivated variables that may influence therapeutic outcomes. In general, the variables contributing to good therapy response can be grouped into five categories. First, previous research has suggested a significant link of client’s satisfaction with strong, supportive, trustworthy, and collaborative *therapist-client alliance* (5, 6, 8, 9). Second, previous studies implied that *settings of psychotherapy*, including more efficient registration procedures, appropriate appointment times and venues, involvement of family members, moderate frequency of interviews, and appropriate session durations, were related to better results in psychotherapy (8, 10, 11). Third, associations between client satisfaction and *personality traits and professional competence of therapists* have also been demonstrated in previous studies. Therapist factors that positively affect the clients’ psychotherapy experience include the therapist’s patience, affinity, enthusiasm, humor, meticulousness, authenticity, professional sensitivity, ability to sort out complex information, empathy, theoretical interpretation, training background of psychiatry, psychoanalytic investigation ability, and facilitative interpersonal skills (5, 8, 12, 13). Fourth, previous research implied that some pretreatment patient characteristics, such as client preferences, severe mental symptoms, depression with less comorbid anxiety, middle age, and unwillingness to accept psychotherapy and medication, were correlated with poorer therapy outcomes (5, 6, 14–16). Fifth, therapy theoretical orientations, strategies, and skills have also been found to be important factors affecting therapeutic outcomes, although conclusions have varied across different studies. Regarding the theoretical orientation of psychotherapy, previous research implied that clients accepting psychodynamic therapy reported more experience with side effects than other treatments, such as family (systemic) therapy, humanistic psychotherapy, and cognitive behavioral therapy (CBT) (17, 18). Concerning therapy skills, in family and psychodynamic therapy, therapeutic techniques and strategies that have shown to be helpful include circular questioning, genograms, homework, visualization techniques, reformulating, metaphor, reflecting team, reframing, promoting individual development, expressing acknowledgment, facilitating emotional flow, self-exploration, and coping with daily practical issues (5, 8, 19–22).

Another issue requiring clarification is the relationship between psychotherapy satisfaction and objective clinical outcomes such as symptom reduction, social function improvement, and increased wellbeing. Psychotherapy satisfaction refers to the client’s positive appraisal of the outcomes and process attributes of a therapy. It is a prominent indicator of the quality of therapy and belongs to the subjective experience of clients (23). Although psychotherapy satisfaction does not necessarily demonstrate a one-to-one relationship with objectively assessed therapy outcomes (24), previous research suggested that they were closely correlated and contributed to each other (25–27). Meanwhile, prior studies have taken both as important indicators for psychotherapy effectiveness (28). However, the mechanism of how these two variables influence each other remains unclear. In the current study, we took psychotherapy satisfaction as an indicator of the client’s therapy effectiveness.

Previous literature suggests that most previous analyses on the predictors of client satisfaction with psychotherapy have been conducted using *a priori* programming of fixed solutions with a specific theoretical hypotheses or through qualitative approaches (29). Only recently have machine-learning approaches been used to predict outcomes of psychotherapy. Machine learning is an emerging area of artificial intelligence that implements a classification or prediction model in a data-driven and no-hypotheses way. To date, studies using machine learning to predict client response to psychological talk therapies can be categorized into two groups. The first cluster of studies includes those predicting psychotherapy outcomes from certain *pretreatment characteristics* of the clients. These characteristics included the client’s demographic, psycho-social and clinical characteristics (e.g., age, ethnicity, gender, economic status, social support, life events, personality trait, the severity of symptoms, and comorbidity), electronic medical records, structured interview data, and brain function (29–34). For example, regarding demographic, psycho-social, and clinical characteristics, Green et al. (35) built a machine-learning model with five pretreatment factors to predict depressed patient’s response to psychotherapy. Those variables included the client’s ethnicity, gender, deprivation, and initial depression and anxiety severity. Their model predicted a reduction of depression symptoms with an accuracy of 74.9% (35). Similarly, Buckman et al. (36) used clinical data such as anxiety and depression symptoms, alcohol use, life events, and social support to predict depressive patients’ remission after 3–4 months of therapy in primary care settings. The prediction power of the nine machine learning models they built was acceptable. Additionally, Gori et al. (37) applied artificial neural network (ANN) technology to analyze the predictive effect of clients’ personality data on their psychotherapy outcome. Their model showed a mean rate of correct classification of 81% in forecasting successful and unsuccessful treatment cases (37). As for brain function, a machine learning analysis on the CBT outcomes of 38 schizophrenic patients implied that psychotic and affective symptom improvement was related to participants’ neural responses to facial affect across frontal-limbic, sensorimotor, and frontal regions (38). A longitudinal study on 49 panic patients who received CBT found that patients’ pretreatment whole brain signals were good predictors of their response to therapy (39).

The second group refers to the process-outcome studies that predicted psychotherapy outcomes based on data *during or in between sessions*, such as theoretical orientations of therapy (e.g., CBT, interpersonal therapy), therapist’s interventions (e.g., therapist’s specific conversation strategies, psychodynamic assessment, and intensity of therapy), therapist-client interactions (e.g., psychotherapy conversation text, smartphone messages, session notes and transcripts, session audio acoustics, and video), client’s real-time response to therapy (e.g., completion of the homework assignment, ambient smartphone data, and biomarkers during a session) (32, 40–44). As for theoretical orientations of psychotherapy, Chekroud et al. (33) suggested that some multivariable modeling methods, such as “personalized advantage index” (PAI), could be used to identify which evidence-based therapy approach (e.g., CBT, interpersonal therapy, and psychodynamic therapy) might be effective in patients with complaints including major depression and post-traumatic stress disorder (PTSD). Regarding therapist’s interventions, in a large-scale study on the discourse of text-message-based psychotherapy conversations, Althoff et al. (45) found that actionable conversation strategies were linked with clients’ higher therapy satisfaction. Similarly, an investigation of 14,899 patients suggested that certain therapist utterances of CBT, such as change methods, were associated with more patient engagement and improvement in symptoms (46). As for therapist-client interactions, Nasir et al. (41) found that couple therapy outcomes were closely related to the behavioral interaction and acoustics of the spoken interactions, such as vocal intonation and intensity, during the therapy sessions. Regarding the client’s real-time response, Wallert et al.’s (40) study applied machine-learning technologies to estimate patients’ adherence to internet CBT for depression and anxiety after myocardial infarction. The strongest predictors included self-assessed cardiac-related fear, sex and the number of words the patient used to finish the homework assignment (40). Meanwhile, Chekroud et al. (33) suggested that a valuable future research direction is to track a patient’s real-time response during treatment (e.g., self-reported outcome/symptom measures) and enter them into a machine learning computer system. Then the computerized system might predict the patient’s improvement trajectories by comparing it to an established clinical database (47).

Generally, the current research using machine learning approach to predict psychotherapy outcome and satisfaction is still preliminary. As described above, client satisfaction with psychotherapy is affected by many factors, including clients’ factors, psychotherapists’ factors, and specific strategic factors. We hope to use a variety of machine-learning methods to study this issue from multiple angles. Meanwhile, to date, the majority of studies predicting clients’ response to psychotherapy using machine learning were from Western countries. Although several studies conducted external validation to test the generalizability of certain machine learning (ML) models (43, 48), there is a lack of studies that build an artificial model to predict Chinese clients’ satisfaction with psychotherapy. Comparatively, the Chinese tradition emphasizes more on individuals’ emotional bonding with their families than Western culture. Meanwhile, Chinese families are more influenced by Confucianism and place more emphasis on an individual’s obedience to authority (5, 49). This implies that some special psychotherapy theories, methods or settings, such as systemic family therapy, psychoeducation and being treated in medical institutes, may act as important contributors to client satisfaction with psychotherapy (5, 6). However, the potential factors that contribute to Chinese clients’ therapy satisfaction and the mechanism by which these underlying factors interact with each other remain unclear. This restriction in research may hinder the development and tailoring of more effective psychotherapy strategies.

Thus, in this study, we collected information from both clients and psychotherapists and applied seven types of machine learning algorithms, as well as biostatistics, aiming to (1) identify the most important factors that affect client satisfaction with psychotherapy and (2) design and implement a classifier based on supervised machine learning to predict whether clients are satisfied with psychotherapy. Based on the results of our study, the classifier can provide a predictive outcome of a specific client’s satisfaction with psychotherapy. Meanwhile, we can improve our treatment strategy to provide clients with more personalized therapy services.

## Materials and methods

### Participants

From 5 July to 28 August, 2021, individuals who had received psychotherapy were recruited *via* the WeChat platform. Each of them was asked to complete an electronic questionnaire using their WeChat account. WeChat is a representative mobile social networking platform in China, with more than one billion users. The inclusion criteria for participants were as follows: (1) received or were receiving psychotherapy; (2) had at least one therapy session in the past 4 months; (3) aged 12–60; and (4) agreed to join the investigation and signed the informed consent. Participants were excluded for the following reasons: (1) being diagnosed with severe physical diseases; (2) unable to understand the questions in the investigation; and (3) severe mental disorders with a risk of self-harm.

### Questionnaire

Based on the purpose of the research and a review of literatures, a questionnaire containing the relevant demographic information of the participants and their therapists, as well as certain characteristics of psychotherapy, was compiled. The questionnaire collected the information from participant’s demographic data (age, gender, ethnic group, marriage status, occupation, education, and family economic status), status of psychotherapy (finished or ongoing), psychotherapist’s gender and age, time of the last session, the form of therapy (individual, group, family/couple, and integrative form), the form of access to psychotherapy (face to face, audio, and video), the location where they received the therapy (welfare organization, medical institutes, commercial counseling agency, school, and other), cost per session, qualification of the therapist, the theoretical orientation of therapy (humanistic therapy, systemic therapy, psychodynamic or psychoanalysis therapy, CBT, integrated therapy, or unclear), number of psychotherapists (how many therapists the participant had seen by the time of survey), order of therapy being reflected on, number of sessions (how many sessions had taken place during the therapy being reflected on), diagnosis by psychiatrists, and whether the participant received medication. The client’s satisfaction with psychotherapy was judged based on the answer to the last question: “In general, are you satisfied with the psychotherapy you received?” “Yes” was classified as being satisfied with the psychotherapy; otherwise, the participant was dissatisfied. Detailed information on each feature is listed in Table 1. A total of 15 participants were invited to complete the initial questionnaire and provided their feedback on the content. The final version of the questionnaire was achieved through revisions based on the feedback.

**TABLE 1:** Features of participants involved in the study.

Feature	Satisfied (*n* = 619)	Unsatisfied (*n* = 172)	Overall (%) (*n* = 791)	*P*-value
**Gender**				
*Female*	291 (47.0%)	97 (56.4%)	388 (49.1%)	0.031*
*Male*	328 (53.0%)	74 (43.0%)	402 (50.8%)	
*Transgender*	0	1 (0.6%)	1 (0.1%)	
Age (mean ± SD)	29.8 ± 8.3	31.3 ± 9.7	31.1 ± 8.6	0.071
Ethnic group				0.638
*Han*	594 (96.0%)	163 (94.8%)	757 (95.7%)	
*Minority*	25 (4.0%)	9 (5.2%)	34 (4.3%)	
Marriage status				0.806
*Single*	197 (31.8%)	54 (31.4%)	251 (31.7%)	
*Single with partner*	97 (15.7%)	26 (15.1%)	123 (15.5%)	
*Married without children*	46 (7.4%)	13 (7.6%)	59 (7.5%)	
*Married with children*	262 (42.3%)	71 (41.3%)	333 (42.1%)	
*Divorced or widowed*	17 (2.8%)	8 (4.6%)	25 (3.2%)	
Occupation				0.0004***
*Enterprise and institution staff*	255 (41.2%)	57 (33.1%)	312 (39.4%)	
*Student*	101 (16.3%)	28 (16.3%)	129 (16.3%)	
*Civil servant*	41 (6.6%)	6 (3.5%)	47 (5.9%)	
*Medical personnel*	14 (2.3%)	10 (5.8%)	24 (3.0%)	
*Teacher*	41 (6.6%)	12 (7.0%)	53 (6.7%)	
*Self-employed*	101 (16.3%)	21 (12.2%)	122 (15.4%)	
*Others*	66 (10.7%)	38 (22.1%)	104 (13.3%)	
Education				0.011**
*Junior high school and below*	29 (4.7%)	12 (7.0%)	40 (5.2%)	
*High school*	126 (20.4%)	30 (17.4%)	156 (19.7%)	
*Undergraduate*	409 (66.1%)	101 (58.7%)	510 (64.5%)	
*Master and Ph.D. degree*	55 (8.8%)	29 (16.9%)	84 (10.6%)	
Family economic status				0.160
*Poor*	19 (3.1%)	10 (5.8%)	29 (3.7%)	
*Ordinary*	419 (67.7%)	119 (69.2%)	538 (68.0%)	
*Good*	181 (29.2%)	43 (25.0%)	224 (28.3%)	
Status of psychotherapy				0.179
*Ongoing*	425 (68.7%)	101 (58.7%)	526 (66.5%)	
*Finished*	194 (31.3%)	71 (41.3%)	265 (33.5%)	
Gender of psychotherapist				0.153
*Female*	370 (59.8%)	92 (53.5%)	462 (58.4%)	
*Male*	247 (39.9%)	78 (45.3%)	325 (41.1%)	
*Transgender*	2 (0.3%)	2 (1.2%)	4 (0.5%)	
Age of psychotherapist				0.039*
< *30*	75 (12.1%)	19 (11.0%)	94 (11.9%)	
*30–40*	364 (58.8%)	90 (52.3%)	454 (57.4%)	
*40–50*	159 (25.7%)	49 (28.5%)	208 (26.3%)	
≥ *50*	21 (3.4%)	14 (8.1%)	35 (4.4%)	
Time of the last session				0.128
*Last week*	169 (27.3%)	37 (21.5%)	206 (26.0%)	
*1 week*∼*1 month*	252 (40.7%)	67 (39.0%)	319 (40.3%)	
>*1 month*	198 (32.0%)	68 (39.5%)	266 (33.7%)	
Form of psychotherapy				0.811
*Individual therapy*	411 (66.4%)	116 (67.4%)	537 (67.9%)	
*Group therapy*	19 (3.1%)	7 (4.1%)	26 (3.3%)	
*Family/couple therapy*	139 (22.5%)	35 (20.3%)	174 (22.0%)	
*Integrative therapy*	40 (6.5%)	14 (1.8%)	54 (6.8%)	
Form of access to psychotherapy				0.003**
*Video*	43 (7.0%)	27 (15.7%)	70 (8.8%)	
*Audio*	94 (15.2%)	27 (15.7%)	121 (15.3%)	
*Face-to-face*	358 (57.8%)	82 (47.7%)	440 (55.6%)	
*Mixed*	122 (19.7%)	34 (19.8%)	156 (19.7%)	
*Others*	2 (0.3%)	2 (1.1%)	4 (0.5%)	
Location of psychotherapy				0.002**
*Welfare organization*	31 (5.0%)	16 (9.3%)	47 (6.0%)	
*Hospital*	201 (32.5%)	44 (25.6%)	245 (31.0%)	
*School*	61 (9.8%)	19 (11.0%)	80 (10.1%)	
*Commercial counseling agency*	309 (50.0%)	79 (45.9%)	388 (49.0%)	
*Others*	17 (2.7%)	14 (8.2%)	31 (3.9%)	
Cost per session (mean ± SD)	460.5 ± 810.5	643.9 ± 1,142	500.4 ± 895.5	0.050*
Qualification of the psychotherapist				0.735
*School teacher*	47 (7.6%)	12 (7.0%)	59 (7.5%)	
*Psychologist*	308 (49.8%)	92 (53.5%)	400 (50.6%)	
*Psychotherapist*	195 (31.5%)	45 (26.2%)	240 (30.3%)	
*Social worker*	14 (2.2%)	4 (2.3%)	18 (2.3%)	
*Psychiatrist*	54 (8.7%)	19 (11.0%)	73 (9.2%)	
*No qualification*	1 (0.2%)	0	1 (0.1%)	
Theoretical orientation of psychotherapy				0.042*
*Humanistic therapy*	51 (8.2%)	14 (8.1%)	65 (8.2%)	
*Systemic therapy*	111 (17.9%)	19 (11.1%)	130 (16.4%)	
*Integrated therapy*	66 (10.7%)	18 (10.5%)	84 (10.6%)	
*Psychodynamic or psychoanalysis*	247 (39.9%)	90 (52.3%)	337 (42.6%)	
*Cognitive behavioral therapy*	134 (21.7%)	27 (15.7%)	161 (20.4%)	
*Unclear*	10 (1.6%)	4 (2.3%)	14 (1.8%)	
Number of psychotherapists (mean ± SD)	2.4 ± 1.6	2.4 ± 1.9	2.4 ± 1.7	0.961
Order of the therapy (mean ± SD)	2.2 ± 1.9	2.1 ± 1.1	2.2 ± 1.8	0.439
Number of sessions (mean ± SD)	9.4 ± 23.1	14.9 ± 29.8	10.6 ± 24.8	0.027*
Diagnosis by psychiatrist				0.140
*Yes*	308 (49.8%)	74 (43.0%)	382 (48.3%)	
*No*	311 (50.2%)	98 (57.0%)	409 (51.7%)	
Receiving medicine				0.110
*Yes*	177 (28.6%)	38 (22.1%)	215 (27.2%)	
*No*	442 (71.4%)	134 (77.9%)	576 (72.8%)	

Satisfied, participants satisfied with psychotherapy; unsatisfied, participants unsatisfied with psychotherapy. *P < 0.05 was considered statistically significant; **P < 0.01; ***P < 0.001.

### Procedure

The questionnaire was shared and distributed *via* the WeChat platform. When clicking on the online questionnaire, participants first read a brief introduction about the investigation, such as the aims of the study and the inclusion and exclusion criteria. Then, they decided whether to participate in the survey. The participants were asked to agree and click “yes, I agree to join this investigation” to indicate their informed consent before starting the questionnaire. It was an anonymous investigation. WeChat users or participants who joined the investigation were encouraged to share the investigation in their WeChat moments. They were also asked to forward the investigation to other WeChat groups that they belonged to and to share the questionnaire with their WeChat friends including clients, psychotherapists, psychiatrists, social workers, and schoolteachers. The investigation was accomplished by the participants using either the mobile app or the PC-based interface of WeChat. The completion time for the whole survey was about 5 min. Every participant could complete the survey only once. This research complied with the American Psychological Association Code of Ethics and was approved by the Institutional Review Board at Shanghai Pudong New Area Mental Health Center, Tongji University School of Medicine. Informed consent was obtained from each participant.

### Modeling using machine learning

**FIGURE 1:**
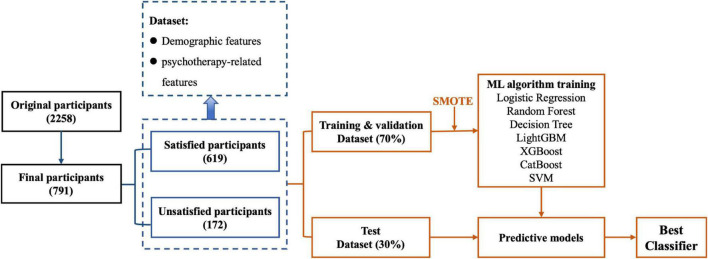
The flowchart of data processing and machine learning-based modeling. The raw dataset was processed by removing non-compliant data entries to form the dataset used in the study. The dataset consisted of participants’ demographic features, and psychotherapy-related features were split into a training and validation dataset and a test dataset. Different machine learning algorithms were selected for training based on the training and validation dataset. Predictive models were obtained after parameter tuning. The final classifier was determined according to the comparison of each trained model’s prediction performance using the test dataset.

In this study, we leveraged machine-learning technologies to predict participants’ satisfaction with psychotherapy and evaluated the predictive performance of training models. Our machine-learning-based modeling process has four key steps, namely, pre-processing of raw data, selection of features, selection of algorithms, and tuning of the parameters. Finally, we compare the predictive performance of all the models and choose the best classifier. Figure 1 shows the detailed workflow. The method was also described in our previous study (50). Scikit-learn 1.1.3, ^1^a well-known machine-learning library based on the Python language, was applied to train prediction models (51).

### Pre-processing of raw data and selection of features

In our dataset, 619 participants were satisfied- and 172 participants were unsatisfied with psychotherapy (Table 1). We select 30 features according to the mutual information, and use L1 normalization to pre-process the features. We randomly split the whole dataset into one training/validation subset and one test subset (Figure 1). We used 70% of the participants’ data for training and validation, and used the remaining 30% of the participants’ data for testing (52–54). The training/validation subset was used to both train and validate the prediction model, and the test subset was used to evaluate the performance of the model. To address the problem of unbalanced samples, for the training/validation subset, we used the synthetic minority oversampling technique (SMOTE) approach (55) to oversample the minority type of participants. We further applied the five-fold cross-validation approach to prepare the training/validation subset, where the training/validation subset was randomly divided into five groups of equal size. Of the five groups, one group was retained as the validation data to evaluate the model, and the remaining four groups were used for training. We repeated the cross-validation procedure five times, and each of the five groups was used once for validation.

The selected features were designed to reflect the different aspects of participants who underwent psychotherapy. In this study, our features included the demographic information of the participants and information related to psychotherapy. These features are either numerical or categorical. Table 1 describes the selected features in detail.

### Selection of the algorithm and parameter tuning

To obtain the best prediction model, we selected classical algorithms to implement supervised machine learning, such as logistic regression, decision tree, random forest, and support vector machine (SVM), as well as some emerging approaches, including LightGBM, XGBoost, and CatBoost. For each of these selected algorithms, we aimed to find a “best” parameter set. Based on the training/validation subset, we swept through the parameter space using the grid search approach. We selected a set of possible values of each parameter to form the parameter space. The grid search iterated through each combination of parameters. For each parameter combination, we calculated the prediction performance. To avoid bias, we apply a nested approach, i.e., we repeat the random split for training/validation subset and the test subset for 10 times, and record the average prediction performance. In the end, the parameters leading to the best average prediction performance will be recorded. Our model can now be used to judge a new client’s satisfaction with psychotherapy based on the input information.

### Evaluation of the model performance

To quantify the predictive performance of the trained models, we adopted the following three classic metrics: precision, recall, and F1 score (56). Precision indicates the fraction of the model comprising participants who actually satisfied with their psychotherapy. Recall indicates the fraction of participants with psychotherapy satisfaction who have been correctly uncovered by the model. The F1 score represents the harmonic mean of the precision and the recall metric. The best F1 score is 1, and the worst is zero. A higher F1 score indicates better predictive performance of a model.

### Statistics

We implemented statistical analysis using the Python programming language. The numerical variables were represented in the form of the mean ± standard deviation (SD) (Table 1); categorical variables were shown as numbers and percentages. *P*-values in Table 1 were obtained by using the chi-square test (57). A *p*-value less than 0.05 was considered statistically significant. Chi-square (χ^2^) statistics were used to quantify the dependence of each selected feature and the groups of participants (satisfied or dissatisfied with psychotherapy) (57). A larger χ^2^ value indicates that a feature has higher discriminative power.

## Results

### The demographics of the participants

In total, 925 participants completed the original questionnaire. By removing non-compliant data entry, the information of 791 (85.5%) participants were finally analyzed in our study. In the dataset, 619 participants reported that they were satisfied with the psychotherapy they received, while 172 participants expressed dissatisfaction with the therapy (Table 1). The incidence of participants who were satisfied with psychotherapy was 78.3%. Each participant’s data contained 22 main features, which can be numerical (such as age, cost per session, number of therapies received, order of therapy being reflected on, and number of sessions) or categorical (other features). Detailed information on the participant number, percentage, and *p*-value of each feature is shown in Table 1.

**FIGURE 2:**
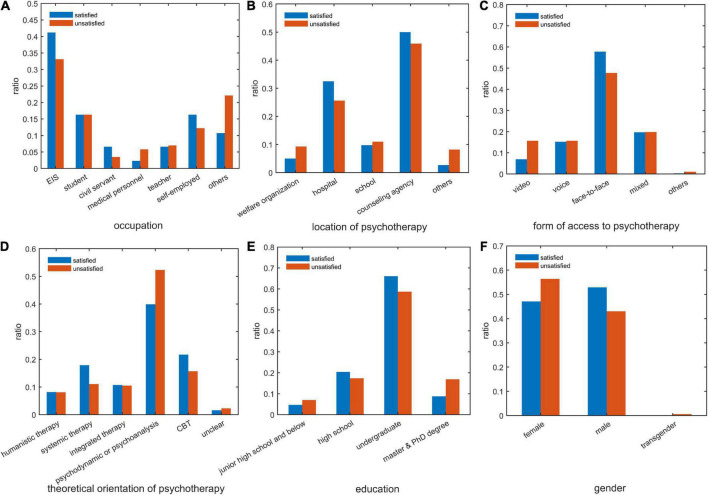
Comparison of clients’ satisfaction with psychotherapy based on graph metrics. **(A)** client’s occupation; **(B)** location of psychotherapy; **(C)** way of psychotherapy; **(D)** theoretical orientation of psychotherapy; **(E)** client’s education; **(F)** client’s gender. satisfied: participant satisfied with psychotherapy; unsatisfied: participant unsatisfied with psychotherapy. EIS, enterprise and institution staff; CBT, cognitive behavioral therapy.

A total of 328 (53.0%) male participants were satisfied with the therapy they received, while fewer female participants (291, 47.0%) were satisfied (*p* = 0.031, see also Figure 2F). The average cost per session of the unsatisfied group was much higher than that of the satisfied group (643.9 ± 1,142 vs. 460.5 ± 810.5, *p* = 0.05). Regarding the number of sessions underwent by the participants, the average number of sessions in the unsatisfied group was much higher than that in the satisfied group (14.9 ± 29.8 vs. 9.4 ± 23.1, *p* = 0.027). Besides, features, such as occupation, education, psychotherapist’s age, method of psychotherapy, therapy location, and psychotherapy theoretical orientations, were significantly different between the two groups of participants (Table 1).

### Important features distinguishing clients who were satisfied or unsatisfied with psychotherapy

Next, chi-square analysis was used to evaluate each feature’s discriminative power for the categories of clients who were satisfied or unsatisfied with psychotherapy. The top 10 features that most contributed to distinguishing clients’ psychotherapy satisfaction include occupation, therapy location, form of access to psychotherapy, theoretical orientation of psychotherapy, education, gender, psychotherapist’s age, psychotherapy status, time of the last session, and psychotherapist’s gender, with chi-square values of 24.913, 16.856, 16.046, 11.490, 11.134, 8.645, 8.370, 5.530, 4.116, and 3.759, respectively (Table 2).

**TABLE 2:** The ranking of feature importance.

Rank	Feature	Chi-square value
1	Occupation	24.913
2	Location of psychotherapy	16.856
3	Form of access to psychotherapy	16.046
4	Theoretical orientation of psychotherapy	11.490
5	Education	11.134
6	Gender	8.645
7	Age of psychotherapist	8.370
8	Status of psychotherapy	5.530
9	Time of the last session	4.116
10	Gender of psychotherapist	3.759

To visualize the difference between clients satisfied or unsatisfied with psychotherapy, we compared the distribution of the top six features in the feature importance ranking of the two types of clients in Figure 2. The client’s occupation is the feature that most strongly distinguishes between those participants who were satisfied and those who were unsatisfied with psychotherapy. Enterprise and institution staff, civil servants, and self-employed individuals had a higher percentage of psychotherapy satisfaction, while medical personnel and others had a higher percentage of dissatisfaction (Figure 2A). Concerning therapy location, clients of medical institutes, and counseling agencies were more satisfied with therapy, while clients of public welfare organizations and other consulting agencies were relatively less satisfied (Figure 2B). Regarding the form of access to psychotherapy, the patients engaged in face-to-face therapy showed higher satisfaction than the patients receiving psychotherapy by other methods (Figure 2C). In terms of the theoretical orientation of therapy, individuals undergoing systemic family therapy and CBT showed a higher percentage of satisfaction than those in psychodynamic therapy (Figure 2D). Interestingly, clients with an education level of junior high school and below or master’s and doctoral degrees had a lower rate of psychotherapy satisfaction (Figure 2E). Moreover, compared with female clients, male clients were relatively more satisfied with psychotherapy (Figure 2F).

### Machine learning algorithms applied for the prediction of client psychotherapy satisfaction

**FIGURE 3:**
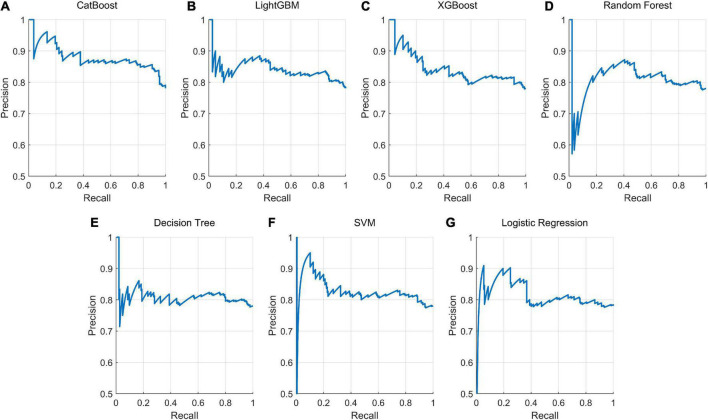
Precision-recall curve for each trained model in predicting client psychotherapy satisfaction. The precision-recall curve for each prediction model predicts whether the participant is satisfied with psychotherapy. Seven machine learning algorithms were selected for training, namely, **(A)** CatBoost, **(B)** LightGBM, **(C)** XGBoost, **(D)** random forest, **(E)** decision tree, **(F)** support vector machine (SVM), and **(G)** logistic regression.

Next, we used a series of supervised machine-learning algorithms to predict clients’ psychotherapy satisfaction. First, we chose seven representative machine-learning algorithms, i.e., CatBoost, LightGBM, XGBoost, random forest, decision tree, SVM, and logistic regression, to build prediction models. Then, based on the test subset, we compared all the models’ predictive performances to find the best prediction model. The F1 scores of the seven models, CatBoost, XGBoost, random forest, LightGBM, SVM, decision tree, and logistic regression, were 0.758, 0.735, 0.734, 0.725, 0.716, 0.701, and 0.612, respectively (Table 3). The precision value and recall value of each model were also listed in Table 3, and the precision-recall curves of the seven models were presented in Figure 3. By comparing these models’ prediction performances, the model based on the CatBoost algorithm achieved the largest F1 score of 0.758, leading to the best performance in predicting client psychotherapy satisfaction.

**TABLE 3:** Compare the performance of different ML algorithms to predict clients’ satisfaction with psychotherapy.

Classifier	Precision	Recall	F1-Score
CatBoost	0.757	0.789	0.758
LightGBM	0.726	0.768	0.735
XGBoost	0.731	0.776	0.734
Random forest	0.725	0.776	0.725
Decision tree	0.715	0.717	0.716
SVM	0.684	0.730	0.701
Logistic regression	0.680	0.578	0.612

## Discussion

To the best of our knowledge, our work is the first study applying machine-learning algorithms to predict clients’ satisfaction with psychotherapy in China. There were two main findings in the current study: (1) the most relevant six features in distinguishing clients with or without satisfaction with psychotherapy were the client’s occupation, location of therapy, the form of access to psychotherapy, theoretical orientation of therapy, client’s education, and gender; (2) the CatBoost algorithm-based model performed the best in distinguishing between satisfied- and unsatisfied participants with psychotherapy, with an F1 score of 0.758. Meanwhile, our study demonstrated the value and feasibility of using machine-learning approaches to predict clients’ psychotherapy satisfaction based on the features of the participants and their therapists.

Among various features determining participants’ psychotherapy satisfaction, occupation was identified as the best feature for distinguishing between those satisfied and those unsatisfied participants with psychotherapy. Our analysis showed that enterprise and institution staff, civil servants, and self-employed individuals had a higher percentage of psychotherapy satisfaction than medical personnel and others (Figure 2A). The reasons for this difference might be related to the different mindsets and educational experiences of different groups. Medical personnel may be more trained in the mindset of biological medicine (1, 58). It may be somewhat at odds with interpretative, speculative, and circular philosophies of psychotherapy (1, 58). This may hinder their engagement with the therapist, and psychotherapy process triggers their dissatisfaction with psychotherapy (6). Hence, for medical personnel, more practical and linear interventions (e.g., linear questioning and action suggestions) might be more applicable (6). However, future research to explore improving medical personnel’s satisfaction with psychotherapy is still needed.

Another finding is that participants with a graduate degree or less than a high school educational level reported more dissatisfaction than those with high school and undergraduate education levels (Figure 2E). Previous research did not strongly support an association between the client’s education level and psychotherapy outcomes (59–61). A possible explanation for this finding might be that for participants with less than a high school education, the process and pragmatic system of psychotherapy may be confusing for them. Therefore, they may receive fewer gains from psychotherapy. By comparison, for participants with high school and undergraduate education levels, their cognitive levels and expectations may be more suitable for therapists in the current study. This might be partially consistent with previous studies, which reported that a higher cognitive level similarity between the therapist and the client could improve the clients’ therapy experience (5). Our finding implies that psychotherapy strategies should be tailored to clients’ cognitive and education levels. However, why participants with a graduate degree had lower therapy satisfaction still needs to be explored in the future.

Concerning the location of psychotherapy, we found that clients who received psychotherapy in mental health institutes, comprehensive hospitals, and commercial agencies were more satisfied with psychotherapy than those whose sessions were conducted at public welfare organizations. The potential factors contributing to this difference may include: (1) Chinese culture advocates worship and trust in authority (5, 49). The participants may think clinicians in “official and professional” medical institutes are more trustworthy. The stereotype of “authorized professionals in official hospitals” may increase participants’ trust in their therapists and adherence to the therapy. This coincides with the findings of previous research that Chinese clients expect their psychotherapists to provide guidance and suggestions from a professional authoritative perspective (5). (2) In China, the admission criteria for psychotherapists in official hospitals and commercial agencies may be stricter than those in public welfare organizations. Clinicians may be more experienced and competent in prescribing various therapy strategies and interventions. In contrast, the percentage of novice therapists and interns in public welfare organizations may be higher (62). Previous studies have also suggested that clients’ therapy outcomes are positively related to the professional competence of therapists (5, 8, 12). (3) In hospitals and commercial agencies, participants need to pay for psychotherapy. Therefore, they may take the therapy more seriously and be more engaged and attentive (63). Meanwhile, they may be more convinced by the therapist’s feedback and suggestions than those participants who receive free treatment in public welfare institutions. Interestingly, our analysis also showed that for participants who were satisfied with their paid sessions, the average cost per session was lower than those who were unsatisfied (Table 1). This finding was in accordance with the study by Stanley et al. (64) that financial incentives that reward therapy attendance with discounted fees were associated with clinical improvement in the clients. It implies that psychotherapy with modest charges may be more helpful. However, the mechanism of this phenomenon and the strategies to solve this problem remain unclear. Future research exploring the strategies (e.g., recruiting more experienced psychotherapists with a medical training background or giving flexible charging policies for specific clients) to improve clients’ satisfaction in welfare organizations is strongly suggested.

In our study, participants who received systemic family therapy and CBT reported a higher percentage of satisfaction than those in psychodynamic therapy (Figure 2D). This finding was partially consistent with previous studies that psychodynamic therapy showed a higher risk of side effects in psychotherapy (6, 17, 18). Psychoanalysis therapy emphasizes the exploration of past traumatic experiences, clients’ defects, and their self-reflection on internal conflicts and pain. It may trigger participants to blame themselves or others for their problems, thus taking the role of an isolated victim and presenting a defect-orientation thinking model (6). Even if participants experience sudden gains from the therapy (65), it may put much pressure on the clients. Comparatively, systemic family therapy focused on the resources and flexibility of the participants’ families. Meanwhile, there is a greater focus on improving and reframing dysfunctional family interactions (5, 6, 66). Chinese culture attaches greater importance to the influence of the family environment on an individual’s mental health, and family interpersonal conflicts are correlated with various clinical complaints of Chinese clients (5, 67–69). Hence, participants who received systemic family therapy may have more positive perceptions of themselves and their families while also experiencing more significant adjustments in their family relationships. This may contribute to the relief of their symptoms and increase their satisfaction with psychotherapy. Previous research has also implied that the involvement of family members was associated with better outcomes in psychotherapy (10). CBT emphasizes finding more effective coping strategies and cognitive schemes to solve clients’ difficulties (30). This therapeutic philosophy may coincide with Chinese culture, which advocates obedience to professional authority, useful knowledge, and effective coping strategies (5, 49). As a result, participants experienced higher therapy satisfaction.

In terms of the association of therapy satisfaction with participants’ gender, the results of previous studies have varied, and no consistent conclusions have been reached (59). For example, both Vitinius et al. (59) and Schneider and Heuft’s (70) studies found that client gender did not have a significant impact on psychotherapy success. Although our analysis implied that male participants were more satisfied with psychotherapy than female participants, more research is strongly suggested to clarify the potential factors and mechanisms contributing to this gender difference. Regarding the form of access to psychotherapy, face-to-face therapy rated higher in satisfaction than other psychotherapy methods. The main reason may be because the flow of emotions, exchange of ideas, and behavioral interactions between clients and therapists are more fluent during offline psychotherapy (9). Thus, a high-quality therapeutic alliance might be more easily fostered. As suggested by previous research, client satisfaction with therapy was positively correlated with supportive and trustworthy therapist-client alliance (5, 9).

Regarding therapists’ age and gender, our analysis implied that they worked as two of the top 10 features that most contributed to distinguishing client psychotherapy satisfaction (Table 2). However, their contributions were relatively lower (with chi-square values of 8.370 and 3.759, respectively) than the other six features mentioned above. As suggested by previous research, clients whose preferences for the therapist’s gender and age were met reported better therapy outcomes (5, 9). However, no linear correlation between client satisfaction and the therapist’s gender and age have been identified by prior studies. Although our results suggested that clients treated by younger therapists were more satisfied with their treatment (Table 1), future research exploring how therapists’ age affects the participants’ satisfaction is still suggested.

Another issue concerns the impact of diagnosis and symptom severity on participant satisfaction. These two features were not involved in our investigation, but previous research implied that their influence on clients’ responses to psychotherapy could be complicated. Some studies suggested that symptom severity and diagnosis would work as predictors for psychotherapy outcomes (71). Meanwhile, previous research implied that symptom severity might also moderate the associations between other predictors and psychotherapy outcomes (72). However, the mechanisms they interact with other potential features influencing Chinese clients’ psychotherapy satisfaction remain unclear. Therefore, future studies exploring the impact of Chinese clients’ diagnosis and symptom severity on their therapy satisfaction are strongly suggested.

Clients seeking psychotherapy always expect satisfactory outcomes. However, due to differences in clients, therapists, and other relevant factors, not all clients will have satisfactory outcomes. Although the psychotherapeutic process is relatively subjective, our attempts show that its outcomes can still be predicted by models. By applying seven different types of supervised machine-learning-based algorithms, our research showed that, for clients undergoing psychotherapy, the model can accurately distinguish between those who are satisfied or unsatisfied with therapy based on the features of participants and therapists. Among the seven models, the CatBoost algorithm-based model showed the best performance in predicting clients’ psychotherapy satisfaction, with an F1 score of 0.758. CatBoost is based on gradient-boosted decision trees developed by Yandex researchers and engineers (73). It has been employed in a wide variety of fields due to its great performance for classification and regression tasks (74). To date, there are few studies using machine-learning methods to predict satisfaction with psychotherapy. Different studies have used different features to predict satisfaction with psychotherapy from different aspects (29). We are the first study to incorporate both client and therapist factors in a model to predict treatment satisfaction. In addition, previous studies often used a single algorithm or a few (37, 40, 41, 75), making the prediction effect relatively limited. Our research used a variety of machine-learning algorithms, including traditional algorithms such as logistic regression, decision tree, random forest, and SVM, also some emerging approaches such as CatBoost, XGBoost, and LightGBM. Different algorithms have different predictive effects due to different principles and computing abilities. By comparing the performance of multiple algorithms and using automatic parameter tuning, the model we trained can achieve the best prediction performance to the greatest extent possible.

The CatBoost-based machine-learning model achieved in the current study is sufficiently accurate and could provide meaningful implications for psychotherapy practice. The CatBoost-based prediction can be implemented as software or app on the mobile device without special equipment or materials. Input the relevant information of the future client and the counselor, and the model can easily and quickly give whether the client is satisfied with the psychotherapy. The results provided by the model can be used as part of an auxiliary diagnosis and treatment. Still, the precise treatment for a specific client requires the therapist to consider both the given by the model and their own experience, then formulate an appropriate plan to improve the effectiveness of psychotherapy.

## Limitations

This study still has some limitations. First, in the current study, the psychotherapy satisfaction of clients was not assessed in an independent survey. This may make our finding less valid. Meanwhile, the objectively measured outcomes (e.g., symptom reduction and improvement in social function) of psychotherapy were not included in the investigation. The relationships between client satisfaction and treatment outcomes were not explored. Hence, randomized control trial and longitudinal research using objective and experimental data will be introduced in the future. Second, the self-assessed questionnaires were disseminated and completed online *via* social media applications according to the inclusion and exclusion criteria. Although our sample size was relatively large, the validity and accuracy of the survey on some variables (e.g., whether participants actually received the therapy as indicated, the age and qualification of the therapist, and the theoretical orientation of therapy) might not be sufficiently guaranteed. Third, some other potential features associated with client satisfaction, such as therapists’ education level, career stage, professional experience and mental activity, competence, participant’s detailed diagnosis, symptom severity, therapy contents, frequency, and treatment quality were not investigated. Future studies using qualitative approaches or quantitative frameworks to explore the underlying mechanisms of how these factors influence client satisfaction with psychotherapy are strongly suggested. Forth, we conducted a binary assessment of psychotherapy satisfaction in the current study. In future studies, we will increase the sample size, conduct a more nuanced classification of psychotherapy satisfaction, and refine the current predictive model.

## Conclusion

The current study clarified several major factors influencing client satisfaction with psychotherapy, including the client’s occupation, gender, education, location of psychotherapy, the form of access to psychotherapy, and theoretical orientation of therapy. It suggests that good therapy strategies should be designed in accordance with the certain demographic characteristics of the clients and their specific preferences for therapy settings and approaches. Meanwhile, we built a supervised machine-learning-based model which could distinguish between satisfied or unsatisfied participants with psychotherapy. The model based on the CatBoost algorithm achieved an F1 score of 0.758. These results provide meaningful implications for designing and tailoring better psychotherapy strategies for specific clients to achieve better therapeutic outcomes.

## Data availability statement

The raw data supporting the conclusions of this article will be made available by the authors, without undue reservation.

## Ethics statement

The study involving human participants was reviewed and approved by the Ethics Committee of Tongji University and the Shanghai Pudong New Area Mental Health Center (No. PWRd2020-01). The patients/participants provided their written informed consent to participate in this study.

## Author contributions

LY and LL substantially contributed to the design, participant recruitment, data analysis, and draft the manuscript. HG was responsible for recruiting data. XZ contributed to the study conception and critical review of the manuscript for content. ZW and YC implemented machine learning algorithms and statistical analysis. All authors contributed to the article and approved the submitted version.

## Footnotes

1
https://scikit-learn.org/stable/

## References

[1] Rybarczyk B. Psychotherapy. In: Kreutzer J DeLuca J Caplan B editors. *Encyclopedia of Clinical Neuropsychology.* Cham: Springer (2018).

[2] Li J Wang X Meng H Zeng K Quan F Liu F. Systemic family therapy of comorbidity of anxiety and depression with epilepsy in adolescents. *Psychiatry Investig.* (2016) 13:305–10. 10.4306/pi.2016.13.3.305 27247596 PMC4878964

[3] Dragioti E Karathanos V Gerdle B Evangelou E. Does psychotherapy work? An umbrella review of meta-analyses of randomized controlled trials. *Acta Psychiatr Scand.* (2017) 136:236–46. 28240781 10.1111/acps.12713

[4] Levitt HM Pomerville A Surace FI. A qualitative meta-analysis examining clients’ experiences of psychotherapy: a new agenda (vol 142, pg 801, 2016). *Psychol Bull.* (2016) 142:1067–1067. 10.1037/bul0000057 27123862

[5] Liu L Wu J Wang J Wang Y Tong Y Ge C What do chinese families with depressed adolescents find helpful in family therapy? A qualitative study. *Front Psychol.* (2020) 11:1318. 10.3389/fpsyg.2020.01318 32714235 PMC7344142

[6] Yao L Zhao X Xu Z Chen Y Liu L Feng Q Influencing factors and machine learning-based prediction of side effects in psychotherapy. *Front Psychiatry.* (2020) 11:537442. 10.3389/fpsyt.2020.537442 33343404 PMC7744296

[7] Liu L Miller JK Zhao X Ma X Wang J Li W. Systemic family psychotherapy in China: a qualitative analysis of therapy process. *Psychol Psychother.* (2013) 86:447–65. 10.1111/j.2044-8341.2012.02075.x 24217868

[8] Løvgren A Røssberg JI Nilsen L Engebretsen E Ulberg R. How do adolescents with depression experience improvement in psychodynamic psychotherapy? A qualitative study. *BMC Psychiatry.* (2019) 19:95. 10.1186/s12888-019-2080-0 30898111 PMC6429792

[9] Flückiger C Del Re AC Wampold Bruce E Horvath Adam O. The alliance in adult psychotherapy: a meta-analytic synthesis. *Psychotherapy.* (2018) 55:316–40. 10.1037/pst0000172 29792475

[10] Dardas LA van de Water B Simmons LA. Parental involvement in adolescent depression interventions: a systematic review of randomized clinical trials. *Int J Ment Health Nurs.* (2018) 27:555–70. 10.1111/inm.12429 29277947

[11] Williams R Farquharson L Palmer L Bassett P Clarke J Clark DM Patient preference in psychological treatment and associations with self-reported outcome: national cross-sectional survey in England and Wales. *BMC Psychiatry.* (2016) 16:4. 10.1186/s12888-015-0702-8 26768890 PMC4714467

[12] Schöttke H Flückiger C Goldberg SB Eversmann J Lange J. Predicting psychotherapy outcome based on therapist interpersonal skills: A five-year longitudinal study of a therapist assessment protocol. *Psychother Res.* (2017) 27:642–52. 10.1080/10503307.2015.1125546 28277042

[13] Anderson T McClintock AS Himawan L Song X Patterson CL. A prospective study of therapist facilitative interpersonal skills as a predictor of treatment outcome. *J Consult Clin Psychol.* (2016) 84:57–66. 10.1037/ccp0000060 26594945

[14] Spinelli MG Endicott J Goetz RR Segre LS. Reanalysis of efficacy of interpersonal psychotherapy for antepartum depression versus parenting education program: initial severity of depression as a predictor of treatment outcome. *J Clin Psychiatry.* (2016) 77:535–40. 10.4088/JCP.15m09787 27137422

[15] Lopes RT Gonçalves MM Sinai D Machado PP. Predictors of dropout in a controlled clinical trial of psychotherapy for moderate depression. *Int J Clin Health Psychol.* (2015) 15:76–80. 10.1016/j.ijchp.2014.11.001 30487824 PMC6224779

[16] Lindhiem O Bennett CB Trentacosta CJ McLear C. Client preferences affect treatment satisfaction, completion, and clinical outcome: a meta-analysis. *Clin Psychol Rev.* (2014) 34:506–17. 25189522 10.1016/j.cpr.2014.06.002 PMC4176894

[17] Leitner A Märtens M Koschier A Gerlich K Liegl G Hinterwallner H Patients’ perceptions of risky developments during psychotherapy. *J Contemp Psychother.* (2013) 43:95–105.

[18] Crawford MJ Thana L Farquharson L Palmer L Hancock E Bassett P Patient experience of negative effects of psychological treatment: results of a national survey. *Br J Psychiatry.* (2016) 208:260–5. 26932486 10.1192/bjp.bp.114.162628

[19] de Leon CGP. “What was i thinking?!’ rhetorical questions as a technique to identify and explore impasses in therapy. *Aust NZ J Fam Ther.* (2018) 39:21–37.

[20] Earl RM. Video game use as a tool for assessing and intervening with identity formation and social development in family therapy. *Aust NZ J Fam Ther.* (2018) 39:5–20.

[21] Larner G. Utilising gaming, rhetorical questions, deception genograms, and other useful techniques in family therapy. *Aust NZ J Fam Ther.* (2018) 39:3–4.

[22] North J Shadid C Hertlein KM. Deception in family therapy: recognition, implications, and intervention. *Aust NZ J Fam Ther.* (2018) 39:38–53.

[23] Sidani S Epstein DR Fox M Collins L. The contribution of participant, treatment, and outcome factors to treatment satisfaction. *Res Nurs Health.* (2018) 41:572–82. 30221779 10.1002/nur.21909

[24] De Smet M Below C Acke E Werbart A Meganck R Desmet M When ‘good outcome’ does not correspond to ‘good therapy’: reflections on discrepancies between outcome scores and patients’ therapy satisfaction. *Eur J Psychother Counsel.* (2021) 23:156–76.

[25] Ring M Gysin-Maillart A. Patients’ satisfaction with the therapeutic relationship and therapeutic outcome is related to suicidal ideation in the attempted suicide short intervention program (ASSIP). *Crisis.* (2020) 41:337–43. 10.1027/0227-5910/a000644 31918584

[26] Viefhaus P Döpfner M Dachs L Goletz H Görtz-Dorten A Kinnen C Parent- and therapist-rated treatment satisfaction following routine child cognitive-behavioral therapy. *Eur Child Adolesc Psychiatry.* (2021) 30:427–39. 10.1007/s00787-020-01528-1 32306088 PMC8019416

[27] Harper-Jaques S Foucault D. Walk-in single-session therapy: client satisfaction and clinical outcomes. *J System Ther.* (2014) 33:29–49.

[28] Keum BT Wang L. Supervision and psychotherapy process and outcome: a meta-analytic review. *Transl Issues Psychol Sci.* (2021) 7:89–108.

[29] van Doorn KA Kamsteeg C Bate J Aafjes M. A scoping review of machine learning in psychotherapy research. *Psychother Res.* (2021) 31:92–116. 32862761 10.1080/10503307.2020.1808729

[30] Lutz W Rubel JA Schwartz B Schilling V Deisenhofer AK. Towards integrating personalized feedback research into clinical practice: development of the trier treatment navigator (TTN). *Behav Res Ther.* (2019) 120:103438. 10.1016/j.brat.2019.103438 31301550

[31] Reggente N Moody TD Morfini F Sheen C Rissman J O’Neill J Multivariate resting-state functional connectivity predicts response to cognitive behavioral therapy in obsessive-compulsive disorder. *Proc Natl Acad Sci USA.* (2018) 115:2222–7. 29440404 10.1073/pnas.1716686115 PMC5834692

[32] Rubel JA Zilcha-Mano S Giesemann J Prinz J Lutz W. Predicting personalized process-outcome associations in psychotherapy using machine learning approaches-A demonstration. *Psychother Res.* (2020) 30:300–9. 10.1080/10503307.2019.1597994 30913982

[33] Chekroud AM Bondar J Delgadillo J Doherty G Wasil A Fokkema M The promise of machine learning in predicting treatment outcomes in psychiatry. *World Psychiatry.* (2021) 20:154–70. 34002503 10.1002/wps.20882 PMC8129866

[34] Lutz W Leach C Barkham M Lucock M Stiles WB Evans C Predicting change for individual psychotherapy clients on the basis of their nearest neighbors. *J Consult Clin Psychol.* (2005) 73:904–13. 16287390 10.1037/0022-006X.73.5.904

[35] Green SA Honeybourne E Chalkley SR Poots AJ Woodcock T Price G A retrospective observational analysis to identify patient and treatment-related predictors of outcomes in a community mental health programme. *BMJ Open.* (2015) 5:e006103. 10.1136/bmjopen-2014-006103 25995234 PMC4442244

[36] Buckman JEJ Cohen ZD O’Driscoll C Fried EI Saunders R Ambler G Predicting prognosis for adults with depression using individual symptom data: a comparison of modelling approaches. *Psychol Med.* (2021). [Epub ahead of print]. 10.1017/S0033291721001616 33952358 PMC9899563

[37] Gori A Lauro-Grotto R Giannini M Schuldberg D. Predicting treatment outcome by combining different assessment tools: Twoward an integrative model of decision support in psychotherapy. *J Psychother Integr.* (2010) 20:251–69.

[38] Tolmeijer E Kumari V Peters E Williams SCR Mason L. Using fMRI and machine learning to predict symptom improvement following cognitive behavioural therapy for psychosis. *Neuroimage Clin.* (2018) 20:1053–61. 10.1016/j.nicl.2018.10.011 30343250 PMC6197386

[39] Hahn T Kircher T Straube B Wittchen H Konrad C Stroehle A Predicting treatment response to cognitive behavioral therapy in panic disorder with agoraphobia by integrating local neural information. *JAMA Psychiatry.* (2015) 72:68–74. 10.1001/jamapsychiatry.2014.1741 25409415

[40] Wallert J Gustafson E Held C Madison G Norlund F von Essen L Predicting adherence to internet-delivered psychotherapy for symptoms of depression and anxiety after myocardial infarction: machine learning insights from the U-CARE heart randomized controlled trial. *J Med Internet Res.* (2018) 20:e10754. 10.2196/10754 30305255 PMC6234350

[41] Nasir M Baucom BR Georgiou P Narayanan S. Predicting couple therapy outcomes based on speech acoustic features. *Plos One.* (2017) 12:e0185123. 10.1371/journal.pone.0185123 28934302 PMC5608311

[42] Wahle F Mobile sensing and support for people with depression: a pilot trial in the wild. *JMIR Mhealth Uhealth.* (2016) 4:e111. 10.2196/mhealth.5960 27655245 PMC5052463

[43] Symons M Feeney GFX Gallagher MR Young RMD Connor JP. Machine learning vs addiction therapists: a pilot study predicting alcohol dependence treatment outcome from patient data in behavior therapy with adjunctive medication. *J Subst Abuse Treat.* (2019) 99:156–62. 10.1016/j.jsat.2019.01.020 30797388

[44] Villmann T Liebers C Bergmann B Gumz A Geyer M. Investigation of psycho-physiological interactions between patient and therapist during a psychodynamic therapy and their relation to speech using in terms of entropy analysis using a neural network approach. *New Ideas Psychol.* (2008) 26:309–25.

[45] Althoff T Clark K Leskovec J. Large-scale analysis of counseling conversations: an application of natural language processing to mental health. *Trans Assoc Comput Linguist.* (2016) 4:463–76. 28344978 PMC5361062

[46] Ewbank MP Cummins R Tablan V Bateup S Catarino A Martin AJ Quantifying the association between psychotherapy content and clinical outcomes using deep learning. *JAMA Psychiatry.* (2020) 77:35–43. 10.1001/jamapsychiatry.2019.2664 31436785 PMC6707006

[47] de Jong K Conijn JM Gallagher RAV Reshetnikova AS Heij M Lutz MC Using progress feedback to improve outcomes and reduce drop-out, treatment duration, and deterioration: a multilevel meta-analysis. *Clin Psychol Rev.* (2021) 85:102002. 10.1016/j.cpr.2021.102002 33721605

[48] Carcone AI Hasan M Alexander GL Dong M Eggly S Hartlieb KB Developing machine learning models for behavioral coding. *J Pediatr Psychol.* (2019) 44:289–99. 30698755 10.1093/jpepsy/jsy113 PMC6415657

[49] Hou J Chen Z. The trajectories of adolescent depressive symptoms: Identifying latent subgroups and risk factors. *Acta Psychol Sinica.* (2016) 48:957–68.

[50] Yao L Cai M Chen Y Shen C Shi L Guo Y Prediction of antiepileptic drug treatment outcomes of patients with newly diagnosed epilepsy by machine learning. *Epilepsy Behav.* (2019) 96:92–7. 31121513 10.1016/j.yebeh.2019.04.006

[51] Pedregosa F Varoquaux G Gramfort A Michel V Thirion B Grisel O Scikit-learn: machine learning in python. *J Machine Learn Res.* (2011) 12:2825–30. 10.5555/1953048.2078195 34820480

[52] Mcdonald A Sasangohar F Jatav A Rao A. Continuous monitoring and detection of post-traumatic stress disorder (PTSD) triggers among veterans: a supervised machine learning approach. *IISE Trans Healthc Syst Eng.* (2019) 9:201–11.

[53] Gonzalez SDP Delgadillo J Lutz W. Predicting early dropout in online versus face-to-face guided self-help: A machine learning approach. *Behav Res Ther.* (2022) 159:104200. 10.1016/j.brat.2022.104200 36244300

[54] Gunther MP Kirchebner J Lau S. Identifying Direct Coercion in a High Risk Subgroup of Offender Patients With Schizophrenia via Machine Learning Algorithms. *Front Psychiatry.* (2020) 11:415. 10.3389/fpsyt.2020.00415 32477188 PMC7237713

[55] Chawla NV Bowyer KW Hall LO Kegelmeyer WP. SMOTE: synthetic minority over-sampling technique. *J Artif Intell Res.* (2002) 16:321–57.

[56] Fawcett T. An introduction to ROC analysis. *Pattern Recognit Lett.* (2006) 27:861–74.

[57] Yang Y Pedersen JOA. Comparative study on feature selection in text categorization. *Proceedings of the Fourteenth International Conference on Machine Learning.* San Francisco, CA: Morgan Kaufmann Publishers Inc (1997). p. 412–20. 10.5555/645526.657137

[58] Feinstein R Heiman N Yager J. Common factors affecting psychotherapy outcomes: some implications for teaching psychotherapy. *J Psychiatr Pract.* (2015) 21:180–9. 25955260 10.1097/PRA.0000000000000064

[59] Vitinius F Tieden S Hellmich M Pfaff H Albus C Ommen O Perceived psychotherapist’s empathy and therapy motivation as determinants of long-term therapy success-results of a cohort study of short term psychodynamic inpatient psychotherapy. *Front Psychiatry.* (2018) 9:660. 10.3389/fpsyt.2018.00660 30564157 PMC6288472

[60] Hiller W Fichter MM Rief W. A controlled treatment study of somatoform disorders including analysis of healthcare utilization and cost-effectiveness. *J Psychosom Res.* (2003) 54:369–80. 12670616 10.1016/s0022-3999(02)00397-5

[61] Swift JK Callahan JL Cooper M Parkin SR. The impact of accommodating client preference in psychotherapy: a meta-analysis. *J Clin Psychol.* (2018) 74:1924–37. 10.1002/jclp.22680 30091140

[62] Wang M Jiang G-R Yan Y-P Zhou Z-Y. The way for certifying and psychotherapists in China. *Chin Ment Health J.* (2015) 29:503–9.

[63] Clark P Sims PL. The practice of fee setting and collection: implications for clinical training programs. *Am J Fam Ther.* (2014) 42:386–97.

[64] Stanley IH Chu C Brown TA Sawyer KA Thomas E. Improved clinical functioning for patients receiving fee discounts that reward treatment engagement. *J Clin Psychol.* (2016) 72:15–21. 10.1002/jclp.22236 26613565

[65] Aderka IM Kauffmann A Shalom JG Beard C Björgvinsson T. Using machine-learning to predict sudden gains in treatment for major depressive disorder. *Behav Res Ther.* (2021) 144:103929. 10.1016/j.brat.2021.103929 34233251

[66] Sexton TL. Functional family therapy: an evidence-based, familyfocused, and systemic approach for working with adolescents and their families. In: Fiese BH editors. *APA handbook of contemporary family psychology: family therapy and training.* Washington DC: American Psychological Association (2019).

[67] Wang JK Zhao XD. Family functioning and social support for older patients with depression in an urban area of Shanghai, China. *Arch Gerontol Geriatr.* (2012) 55:574–9. 10.1016/j.archger.2012.06.011 22770710

[68] Chen W Huang Y Riad A. Family environment and depression: a population-based analysis of gender differences in rural China. *J Fam Issues.* (2014) 35:481–500.

[69] Lui M Lau G Tam V Chiu H Li S Sin K. Parents’ impact on children’s school performance: marital satisfaction, parental involvement, and mental health. *J Child Fam Stud.* (2020) 29:1548–60. 10.1186/s12913-016-1423-5 27409075 PMC4943498

[70] Schneider G Heuft G. Operationalized psychodynamic diagnosis system and outcome of psychodynamic inpatient psychotherapy in male and female patients. *Zeitschrift Fur Psychosomatische Medizin Und Psychotherapie.* (2018) 64:281–97. 30829160 10.13109/zptm.2018.64.3.281

[71] Simon NM Otto MW Worthington JJ Hoge EA Thompson EH Lebeau RT Outcome prediction of cognitive behaviour therapy for panic disorder: initial symptom severity is predictive for treatment outcome, comorbid anxiety or depressive disorder, cluster c personality disorders and initial motivation are not. *Behav Cogn Psychother.* (2008) 36:99–112.

[72] Seow LLY Page AC Hooke GR. Severity of borderline personality disorder symptoms as a moderator of the association between the use of dialectical behaviour therapy skills and treatment outcomes. *Psychother Res.* (2020) 30:920–33. 32013808 10.1080/10503307.2020.1720931

[73] Prokhorenkova L Gusev G Vorobev A Dorogush A Gulin A. CatBoost: unbiased boosting with categorical features. *Adv. Neural Inf. Process.Syst.* (2018) 31:6639–49. 10.5555/3327757.3327770

[74] Hancock JT Khoshgoftaar TM. CatBoost for big data: an interdisciplinary review. *J Big Data.* (2020) 7:94. 10.1186/s40537-020-00369-8 33169094 PMC7610170

[75] Månsson KN Frick A Boraxbekk CJ Marquand AF Williams SC Carlbring P Predicting long-term outcome of Internet-delivered cognitive behavior therapy for social anxiety disorder using fMRI and support vector machine learning. *Transl Psychiatry.* (2015) 5:e530. 10.1038/tp.2015.22 25781229 PMC4354352

